# Electric Vehicle Smart Charging Reservation Algorithm

**DOI:** 10.3390/s22082834

**Published:** 2022-04-07

**Authors:** Radu Flocea, Andrei Hîncu, Andrei Robu, Stelian Senocico, Andrei Traciu, Baltariu Marian Remus, Maria Simona Răboacă, Constantin Filote

**Affiliations:** 1 Research and Development Department, ASSIST Software, Str. Tipografiei Nr. 1, 720043 Suceava, Romania; radu.flocea@assist.ro (R.F.); andrei.hincu@assist.ro (A.H.); andrei.robu@assist.ro (A.R.); stelian.senocico@assist.ro (S.S.); andrei.traciu@assist.ro (A.T.); remus.baltariu@assist.ro (B.M.R.); 2 Faculty of Electrical Engineering and Computer Science, Stefan cel Mare University of Suceava, Str. Universitatii Nr. 13, 720229 Suceava, Romania; filote@usm.ro; 3 National Research and Development Institute for Cryogenic and Isotopic Technologies—ICSI Rm. Valcea, 240050 Ramnicu Valcea, Romania

**Keywords:** software development, OCPP extension, reservation algorithm, electric vehicle charging, electric vehicle charging management platform

## Abstract

The widespread adoption of electromobility constitutes one of the measures designed to reduce air pollution caused by traditional fossil fuels. However, several factors are currently impeding this process, ranging from insufficient charging infrastructure, battery capacity, and long queueing and charging times, to psychological factors. On top of range anxiety, the frustration of the EV drivers is further fuelled by the uncertainty of finding an available charging point on their route. To address this issue, we propose a solution that bypasses the limitations of the “reserve now” function of the OCPP standard, enabling drivers to make charging reservations for the upcoming days, especially when planning a longer trip. We created an algorithm that generates reservation intervals based on the charging station’s reservation and transaction history. Subsequently, we ran a series of test cases that yielded promising results, with no overlapping reservations and the occupation of several stations without queues, assuring, thus, a proper distribution of the available energy resources, while increasing end-user satisfaction. Our solution is independent from the OCPP reservation method; therefore, the authentication and reservation processes performed by the proposed algorithm run only through the central system, authorizing only the creator of the reservation to start the charging transaction.

## 1. Introduction

Over the last few decades, there has been an alarming increase in the unsustainable exploitation of natural resources and environmental degradation. Nonetheless, a shift toward environmental protection has been registered, with regulation policies being enforced and public awareness steadily increasing.

On a global scale, we are currently witnessing a transition towards low-emission mobility, and it is safe to assume that this process is accelerating. Considering the Paris Agreement, the 2030 Agenda for Sustainable Development and the European Strategy for Low-Emission Mobility [1], governments and industry manufacturers have begun to take consistent steps in implementing these regulations and transitioning to an electro-mobility concept and way of life. Nonetheless, there are several factors that hinder the large-scale adoption of electric vehicles (EV), from the purchasing costs, battery operating range, insufficient charging infrastructure, and long charging times to psychological factors. When studying the challenges and opportunities in adopting electric vehicles, Faizal et al. [2] identified range anxiety, the long queuing time, and the uncertainty of finding an available charging point, when necessary, as some of the factors that negatively impact consumers’ purchasing intentions.

In this context, we propose a solution to enable EV owners to make charging station reservations for upcoming days, particularly for long-distance trips that require multiple charging operations, and to ensure a positive user experience that will ultimately stimulate EV adoption, by keeping queueing times to a minimum and ensuring that EV drivers will reach their destination in roughly the estimated time. Following a review of the literature, we concluded that the issue of EV connector reservation algorithms is not widely debated in the scientific community; therefore, our current paper addresses this gap in literature, proposing a solution that is not dependent on the OCPP reservation method.

To this end, we have structured the present paper into several sections. Following the introduction, the second chapter constitutes a brief overview of the current state of the art concerning congestion prevention, optimal distribution of energy resources, connector reservations, occupancy forecasting, and the use of reservation algorithms as an extension of the OCPP protocol. After presenting the work methodology, we thoroughly describe the custom reservation algorithm and its advantages, compared with the existing OCPP reservation possibilities. To further illustrate the algorithm workflow, we provide, in Section 5, an overview of the Smart EVC platform case study. Finally, we analyze the results and state the conclusion we reached after having conducted this study, as well as its limitations, and the future work envisaged to take the proposed solution a step further.

## 2. State of the Art

As stated in the World Economic Forum’s January 2018 report, “Electric Vehicles for Smarter Cities: The Future of Energy and Mobility” [3], radical sustainable and secure mobility and energy solutions are required to prevent congestion and pollution in urban areas that have already been and will continue to be reshaped by demographic shifts. The authors emphasize that the deployment of charging infrastructure should be based on the long-term mobility transformation. Furthermore, they believe that reducing range anxiety and developing smart charging technologies are critical components of the EV market approach, as they will contribute to the adoption of electro-mobility.

One of the major drawbacks of adopting electromobility, according to Ruzmetov et al. [4], is the EV driver’s lack of certainty that there will be an available charging point once they reach the charging station on their route. The authors present a platform designed to ensure continuous cooperation among the various entities involved: energy suppliers, charging stations, EVs, and EV users, proposing an optimization of EV scheduling and allocation to charging stations. When proposing a charging station, the driver’s destination and the battery level are considered to ensure that they are not diverted from the route. One of the major impediments to widespread EV adoption is the scarcity of fast chargers, ranking third, after price and driving range, according to a survey conducted by the McKinsey Center for Future Mobility. However, with the EV purchasing costs declining and ranges steadily increasing, charging may soon become the most significant barrier to the adoption of electromobility. [5] Finding an available socket has, thus, become a real burden and, while services like ChargePoint or ChargeHub provide consumers with information related to real-time charging point availability, the feature of advance reservations for public charging stations has not been implemented yet [6]. As a result, even if the connector is free when the user accesses one of these platforms, there is a good chance that, in the meantime, another EV has already occupied it, rendering the trip to the charging station based on the platform’s recommendation pointless and contributing, thus, to EV driver’s frustration. Reducing congestion at the charging stations is, therefore, of paramount importance. To this end, a series of studies are dedicated to predicting patterns of charging point occupancy, around which a charging station recommendation system could be built, enabling charging service apps or platforms to optimize the distribution of the available charging resources, while decreasing the end-user’s waiting time [7,8,9,10]. Similarly, Soldan et al. [11] propose using big data stream analysis to enable the optimization of the charging point operations, and correctly forecasting the availability of the charging station. The authors stress the parameters of the predictive model need to be constantly updated, to ensure it provides accurate predictions.

Seitaridisa et al. [12] propose an agent-based simulation system, where the two entities seek to maximise their satisfaction: the EVs’ goal is to maximise the amount of energy charged, while keeping the charging time to a minimum, whereas charging stations strive to optimise the use of available resources and to maximise the number of powered EVs. After EVs send their charging requests to multiple charging stations, they are provided with a schedule or with alternate propositions, in case their initially envisaged schedule cannot be met by the station. The optimal solution is identified using integer linear programming techniques.

Bernal et al. [13] provide a model to optimize charging station management with the use of reservations made in advance, while also calculating the ideal pricing and management strategy, to handle the issue of uncertainty associated with connector availability.

Kumar et al. [14] created a distributed system that, based on the battery level, road conditions, the spatial distribution of charging stations, and their available resources and occupancy levels, plans an energy-efficient route, while employing an agile charging slot reservation approach.

Wang et al. [15], underline the importance of a scheduling strategy to fill the gap between EV charging requirements and the resources provided by the charging stations and to ensure a positive user experience that would encourage EV adoption.

Qin and Zhang [16] conducted a theoretical study that enabled them to develop a distributed scheduling protocol intended to reduce waiting time, which includes both queuing time and actual charging time, during a trip along a highway. The reservations made by EV drivers for their next charging, as well as the reservation adjustments, are based on the minimum waiting time communicated by charging stations, which update this information on a regular basis to allow drivers to make the best choice in terms of waiting time.

Park et al. [17] introduce algorithms for the recommendation of charging stations along a route, ensuring three alternatives for the user, depending on the envisaged final battery level and waiting time.

Cao et al. [18] sought to address a series of issues related to the charging management of EVs travelling longer distances, while ensuring efficiency, scalability, and coordination. The authors propose a publish/subscribe communication system for enabling fast charging, in which network entities share relevant information such as charge point availability and reservations made by the drivers. Furthermore, to coordinate EV drivers’ charging arrangements, they have created a distributed charging management strategy, so as to limit the queuing time by selecting the most appropriate charging station.

In the same line, Hye-Jin et al. [19] propose a scheduling system relying on reservations made by the users, with the main goal of increasing EV user satisfaction by reducing both costs and queuing times, while maximizing charging station utilization. A linear ranking function is constructed around several factors such as estimated time of arrival of the EVs, waiting time, and the energy requirements, in order to facilitate the scheduling process. Badmadjian et al. [20] suggest that reservation schemes could constitute a reliable solution for the challenges faced by the EV industry and infrastructure providers. They propose an architecture for interoperable reservation systems that considers needs of the main network entities involved in the process.

Raboaca et al. [21] also investigated the environmental benefits of electric vehicles and proposed software solutions to encourage the adoption of electric vehicles by providing greater control over the EV charging process. Furthermore, the article provided an OCPP (open charge point protocol) specifications analysis, underlining that the charging process could be improved through the integration of a reservation feature. OCPP is an open standard that enables communication between electric vehicles, charging stations and a central management system, regardless of manufacturer features, and ensures management of all factors and entities involved in the recharging operation [22]. This has ensured that EV networks are open and accessible. As a result, end users benefit from flexibility and the ability to choose which network or charging station they use. The main purpose of OCPP is to ensure the interoperability of the EV charging infrastructure, characterized by versatility and simplicity in terms of use for EV drivers and system administrators, alike [23]. Currently, OCPP 1.6 does not support scheduling the reservation of a connector for a future date, being able to reserve only at the exact moment the user makes the reservation. The reservation is terminated either when the user with the associated idTag arrives at the charging point, the expiry time is reached, or the user cancels the reservation [24].

Oricioni and Conti [25] propose an extension of the OCPP standard that would allow the user to participate in the charging optimization process, introducing an advance reservation option. The authors also point out that OCPP standard does not enable reservations made in advance for a later moment and consider that the underlying reasons for this shortcoming might reside in the complexity of this type of scheduling and the first-come-first-served approach, in the detriment of those with remote reservations.

In this context, the purpose of this paper is to propose a solution to bypass the limitations of the “reserve now” function of the OCPP protocol in terms of advance reservations. Enabling EV drivers to have more control over the charging reservation and guaranteeing that the reserved connector will be available when they reach the charging station, avoiding thus long frustrating queuing times, would ensure a great user experience and encourage the adoption of EVs. The shortcomings of the OCPP reservation feature prevents the users from planning a trip and scheduling, in advance, EV charging operations in one or multiple locations along the route.

The novelty resides in the custom reservation algorithm that addresses this issue and in assuring drivers that the reserved connector will be available when they reach the station, avoiding overlapping reservations.

## 3. Methodology

While conducting research in view of developing the Smart EVC platform—an intelligent charging station management platform based on Blockchain and artificial intelligence—the methodology for the case study in the current paper has been gradually improved, based on the literature (WOS, Science Direct, Scopus), the main keywords applied in the databases being: EV charging scheduling, charging reservation, OCPP reservation, EV smart scheduling. Additionally, the methodology has benefited greatly from the results and feedback received after attending several conferences and technology fairs in the field, including EnergEn 2021—“New Cryogenic and Isotope Technologies for Energy and Environment”, AUTOSTAR 2021—Automotive Open Source System Architecture, and MWC Barcelona 2022—Mobile World Congress.

Therefore, we envisaged a case in which several EV drivers were subjected to long queuing times due to the OCPP reservation protocol that allows for overlapping reservations. To address this issue, we developed an algorithm that generates reservation intervals based on coefficients obtained from the charging station’s reservation and transaction history. We opted to implement the reservation feature as a generic method of planning a reservation for all types of electric vehicles in which drivers are familiar with their vehicles’ specifications and can easily estimate charging time. We performed a series of test cases that yielded promising results, overlapping reservations being completely avoided.

## 4. Custom Reservation Algorithm

As pointed out above, there are several limitations to the OCPP reservation feature, where a reservation is initiated when the driver creates it and is valid until a specified end time, blocking the selected connector for other drivers, except for the one that created the reservation. The reservation lifetime ends when the user starts their transaction [24].

Making this approach possible and following the OCPP path also implies a lot of changes and unnecessary refactoring in the already working code in the Web API for reservations and transactions. Thus, below will present all the downsides of this approach and the benefits of the custom reservation algorithm.

We opted for the implementation of the reservations in Web API because this grants us more control over them. Moreover, the reservation in our system, which will be further developed in the “Use” case section, is made up of both the time span selected by the driver and the time calculated by the algorithm and added to the initial user-set StartTime. Furthermore, this kind of reservation can be made in advance and can be successfully used in the “plan a trip” feature, which provides the user with advance information related to the charging needs and options along the route, enabling them to make reservations for the upcoming trip. To better illustrate the flexibility of this type of reservation, we should underline that it can be easily manipulated by changing the status of the reservation and the connector, according to the user’s activity. Furthermore, a transaction can be automatically interrupted when the reservation comes to an end.

**Figure 1:**
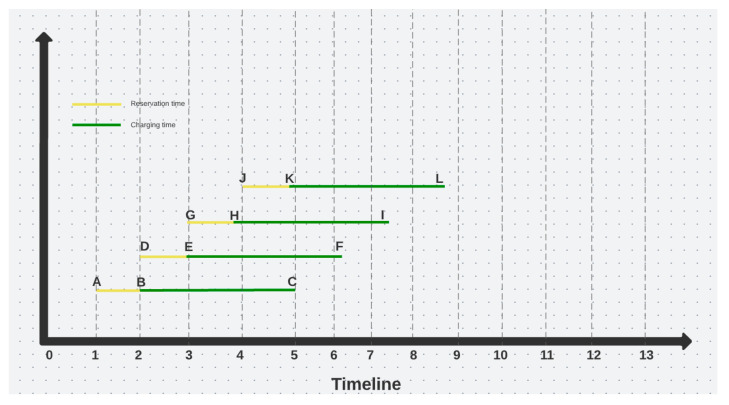
OCPP Reserve now.

**Figure 2:**
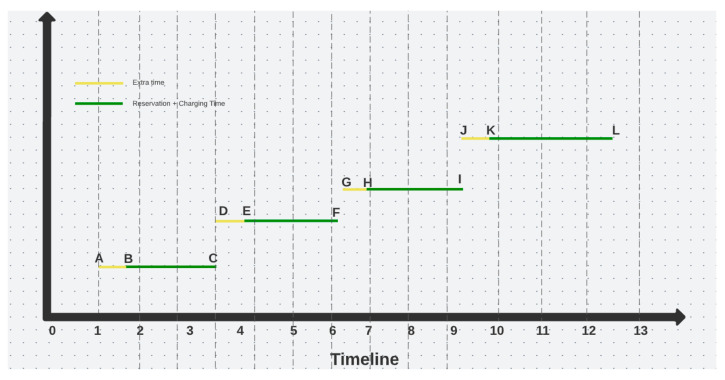
Custom Reservation Algorithm Reservation.

For a better understanding of the differences between the OCPP Reservations and the proposed custom reservation algorithm, the following plot diagrams in Figure 1 and Figure 2 illustrate the significant benefits of the proposed solution.

For the diagram in Figure 1, the *x* axis represents a full day timeline, the *y* axis is only used for a better view and convenience. As mentioned before, in OCPP, the reservation time span is limited to the time before the charging process starts. As illustrated above, the AB, DE, GH and JK segments constitute the reservations. None of the reservations overlap with one another, and this might be considered an optimal solution. Nonetheless, as the users have no limitations imposed by the OCPP reservation, they can block the charging point for as long as they need, and therefore, their charging time is likely to overlap with other reservations. Consequently, this will generate confusion among the other users that made a reservation, and the queueing time will automatically increase.

Alternatively, by using the proposed custom Reservation Algorithm, the diagram and the associated reservations timeslots will look like illustrated below, in Figure 2.

With the custom reservation algorithm, the reservation time is made up of the extra time prior to the charging process and the actual charging time (See Figure 2). Therefore, the reservation of the connector starts at the time selected by the user and ends when the user interrupts the charging process and leaves the station. In this case, there will be no overlaps or uncertainties because the reservation guarantees the availability of the charging point, preventing other users from blocking it during the reservation period. This constitutes the main advantage of the proposed custom reservation algorithm, in comparison with the OCPP Reservations.

To further explain the way in which the proposed custom algorithm works, we have provided below a detailed description of the process.

When making a reservation, the user must select a valid time slot, during which other EV owners will be prevented from charging their vehicles to the reserved charging point. Several status types have been assigned to a charging point reservation: it is “New”, upon creation and then it turns to “In progress” when the reservation interval starts and to “Finished” at the end of the time slot. If the user cancels the reservation, its status becomes “Cancelled”.

Before making a reservation, the user must be assured that no other EVs are or will be plugged to the connector they are about to reserve. For this reason, each reservation is processed by an algorithm designed to determine the best possible StartTime. This algorithm considers several factors and, as a result, will return a new reservation with additional time added to the user-specified StartTime. In the current version, this additional time can be as little as 0 h and as much as 4 h, and it is calculated based on several factors, as follows:

1. All the transactions made in the previous two weeks on the charging point the user is about to reserve are retrieved from the database; these are the only data relevant for the algorithm at this point.
2. Next, two coefficients influencing the output of the algorithm are calculated.

The first one is the connectorRequestCoefficient, which determines the exploitation degree of the connector (charging point), comparing the values from the previous week and two weeks prior. This coefficient influences 50% of the result of the algorithm, and is calculated using to the following formula:

connectorRequestCoefficient = (double)lastWeekTransactions/lastTwoWeeksTransactions

where lastWeekTransactions represents the number of transactions recorded in the previous week on the connector reserved by the user and lastTwoWeeksTransactions returns the number of transactions registered during the last two weeks. According to the formula, this coefficient can range from 1 to 0. More precisely, it can have the minimum value 0 if in the last week there were no transactions on that charging point and all the transactions on that connector occurred only two weeks prior. The maximum value 1 will be returned if all the transactions on that specific charging point occurred in the previous week.

The second coefficient is called overlappingCoefficient and it illustrates the utilization degree associated with the time slot selected by the user. It is calculated similarly to the first coefficient: with the data on the transactions performed in the previous two weeks, we determine how many occurred in a period overlapping the time slot selected by the user. To this end, we created the isOverlapping method, which determines whether two time slots have at least one point in common. The method returns the minimum value 0 when none of the transactions made on the reserved connector in the last two weeks intersect with the selected time slot and the maximum value 1 if all the transactions overlap with this period. This coefficient has a 50% weight in the result of the algorithm.

3. Having made all the calculations and retrieved the connectorRequestCoefficient and the overlappingCoefficient, the final coefficient will be calculated using the formula below, which assigns the same weight to the first two coefficients:

0.5 × connectorRequestCoefficient + 0.5 × overlappingCoefficient

This final coefficient has a value ranging from 0 to 1 and determines how long the reservation will be extended for. The number of extra hours that will be added to the reservation StartTime will be calculated as illustrated below:

hoursToAdd = finalCoefficient × maxExtraHours

We have considered that, in the process, a reservation can be moved from one day to another: for instance, if the reservation is initially made for 01:00 a.m. and the algorithm concludes that two extra hours are necessary, then the reservation will be extended, starting from 11:00 p.m., the previous day.

**Figure 3:**
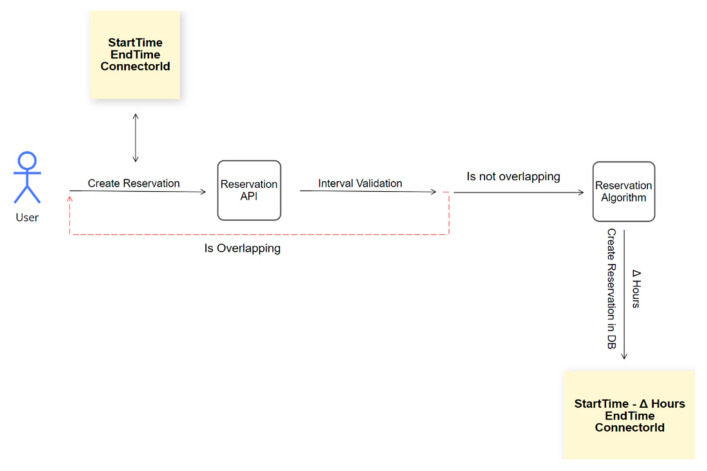
Reservation process.

We have provided in Figure 3 the visual representation of the reservation process executed by the custom reservation algorithm.

To sum up, the users send their reservation request using the SmartEVC platform, thoroughly described in the Case Study section, which is passed through the Reservation API designed to validate the timeframe selected by the user. If no overlapping reservations are detected during the interval validation process, the reservation algorithm establishes the optimal StartTime and EndTime of the reservation, creating the reservation in the database with the suitable ConnectorId. Otherwise, the user will be required to restart the reservation process (Figure 3).

## 5. Case Study

The proposed algorithm has been integrated in the Smart EVC solution—an intelligent charging station management platform based on Blockchain and Artificial Intelligence that allows users to interact with charging stations. The platform brings together various techniques for connecting electric vehicles to charging stations and the embedded Blockchain technology allows for the unification of payment systems for charging electric vehicles.

The mobile app developed within the Smart EVC project will be available on Android and iOS and will enable users to make reservations at the charging stations registered in the system. Using various pre-established parameters, the mobile application facilitates the users’ interactions with charging stations and generates intelligent alerts on upcoming charging options.

To enable users to identify and get directions to available charging stations in their vicinity, the Maps SDK for Android and iOS has been integrated and the Directions and Places APIs from the Google console have been activated.

**Figure 4:**
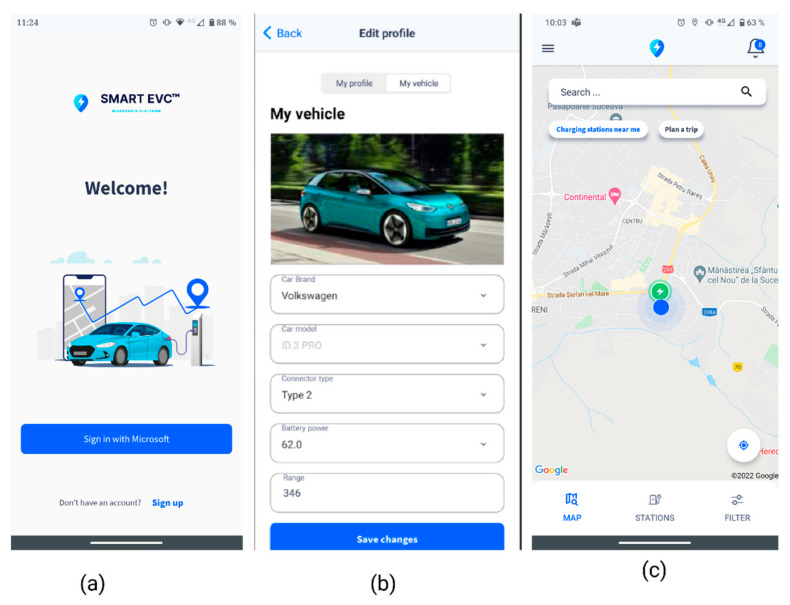
Smart EVC login, profile, and first page. (**a**) login page, (**b**) profile page, (**c**) map with current location and nearby charging stations.

Upon registration to the platform (Figure 4a), the user will be directed to the user and vehicle profile section, where the users can define their profiles. In the current version of our prototype, the user is required to manually input all the information pertaining to the vehicle: brand, model, connector type, battery power, and range. The vehicle image will be automatically inserted from the back end, according to the user input (Figure 4b). Following the user’s authentication into the platform, they will be directed to the MAP screen (Figure 4c). The interaction with the map has been enabled through the LocationListener, GoogleMap.OnMarkerClickListener functions of the Google Maps SDK interfaces.

**Figure 5:**
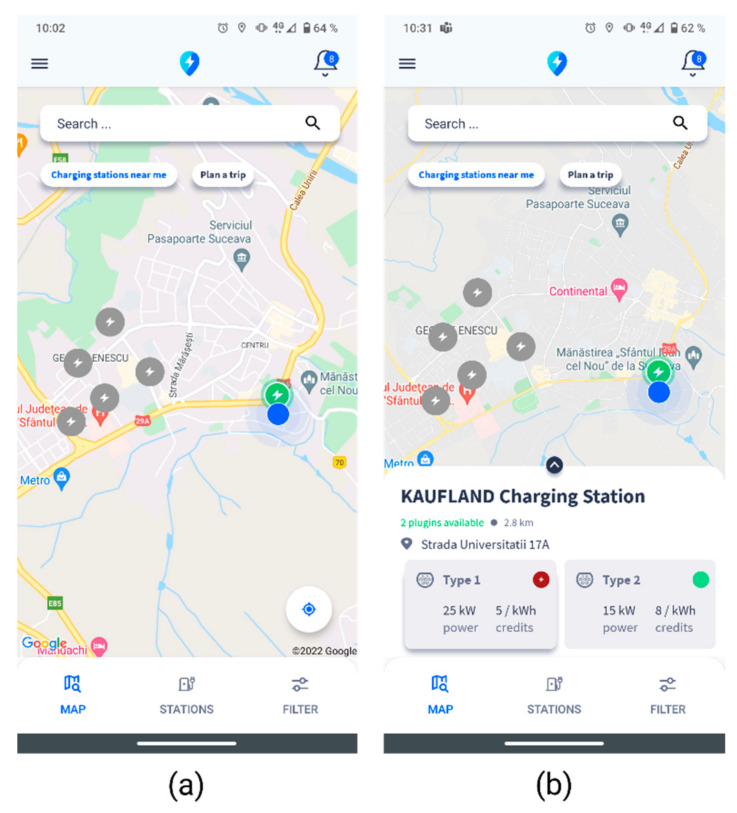
Smart EVC—Charging stations in the vicinity. (**a**) map with charging stations in the vicinity, (**b**) details of a charging station if selected on the map.

By clicking the “Charging stations near me” button, the stations corresponding to the options set by the user in the “Filter” section of the mobile app will be displayed, as illustrated in the Figure 5. The user will click on a marker on the map to view the full details of a charging station: address, distance to the location, and available connectors (Figure 5b).

**Figure 6:**
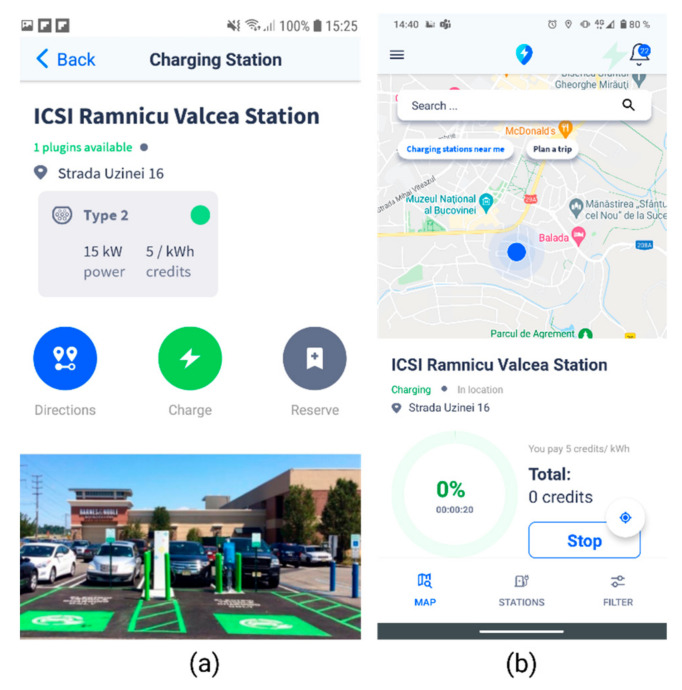
Smart EVC—Charging station. (**a**) full details of a charging station with directions, start charging and reservation options. (**b**) App screen when charging is in progress, with details concerning credits, power level and a Stop transaction button.

Moreover, the user can then expand the view of the station, gaining access to the full functionalities of a station: directions to the station, buttons to initiate the charging process or to make a reservation, as shown in Figure 6a. When the charging transaction is initiated, the user will be provided with information concerning the charging status and the total number of credits that will be withdrawn of their wallet, which is stored in blockchain.

**Figure 7:**
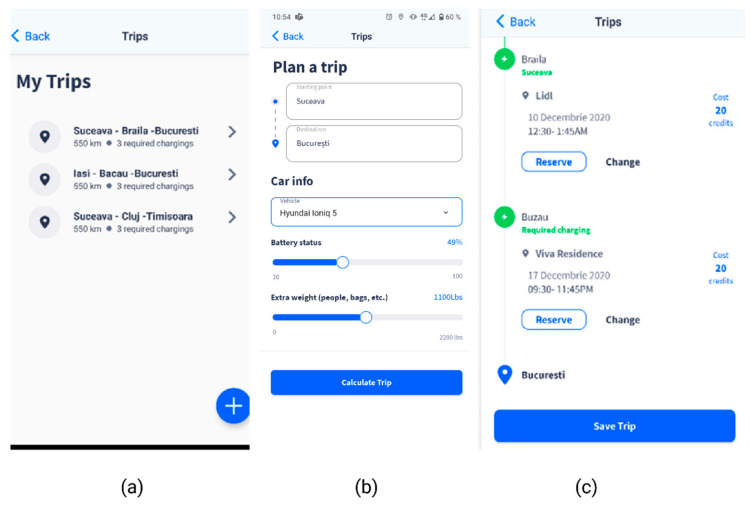
Smart EVC—Plan a trip section. (**a**) EV trips list; (**b**) Plan a trip feature, with details on the battery level and extra weight, based on which the necessary recharging operations are calculated; (**c**) Proposed charging stations along the route, with reservation options.

When the user reaches the station and initiates the charging process, the API called SmartEvcOcppAPI sends other users the busy status, until the charging is complete. The Start/Stop charging transactions are enabled through the unique charging point identifiers: chargingStationId, connectorId, organizationId. The “Plan a trip” section (Figure 7) has been created to assist users in planning their trip, especially for longer routes that require several charging operations, providing the option to make reservations in advance. The particularities of the vehicle for that specific trip, in terms of battery status and extra weight, need to be entered into the platform, to ensure a greater computation accuracy (Figure 7b). The itinerary and the points along the route where charging operations will be required will be displayed, based on the estimates and calculations, as shown in Figure 7c, along with the charging facilities, associated costs, and the “Reserve” option.

**Figure 8:**
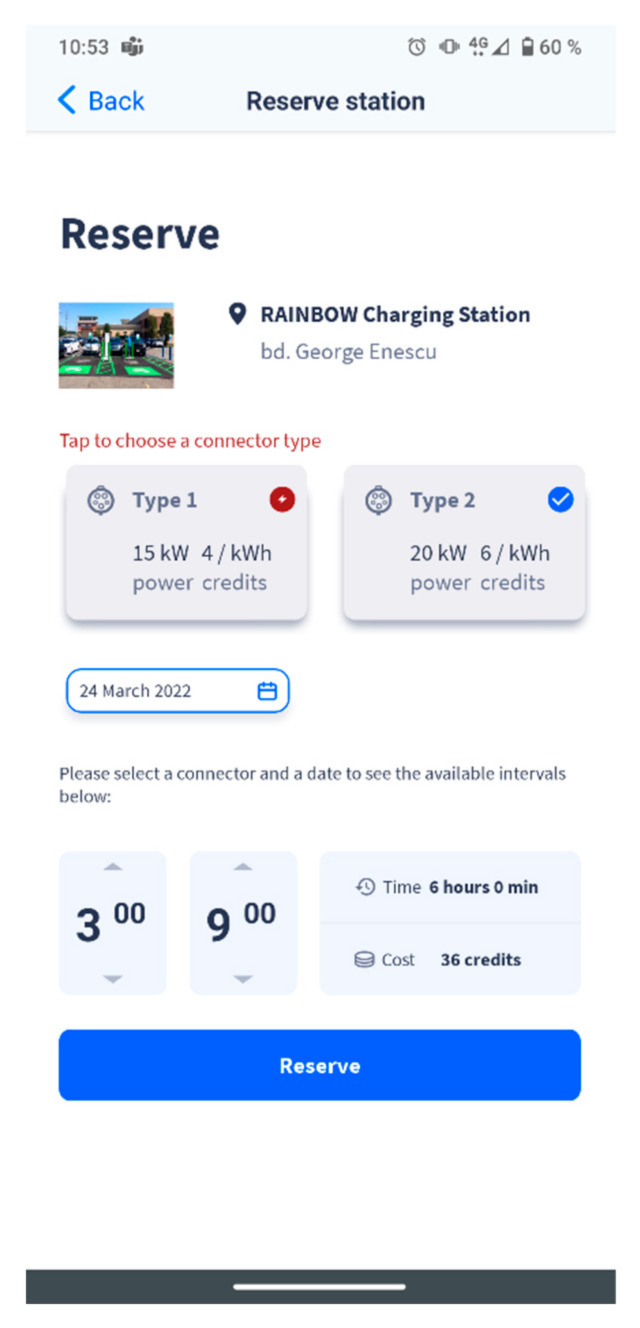
Smart EVC Reservations.

The custom reservation algorithm presented in the current paper has been integrated in the Smart EVC platform, and the EV driver will engage with it when accessing the “Reserve station” page in the mobile application, illustrated in Figure 8.

The users must select the charging station that they want to reserve, as well as a suitable connector for their electric vehicle (Figure 8). Next, they must indicate the date of the reservation and finally, to select a time slot from all the available intervals that will be displayed on the screen. The duration of the reservation and the cost will be automatically calculated as illustrated above.

This is all the data requested from the users, and when they click on the “Reserve station” button, the reservation algorithm will be applied on the data provided by the user, and the StartTime will be adjusted based on all the important factors and statistics on the chosen station, connector, timeline, etc.

**Figure 9:**
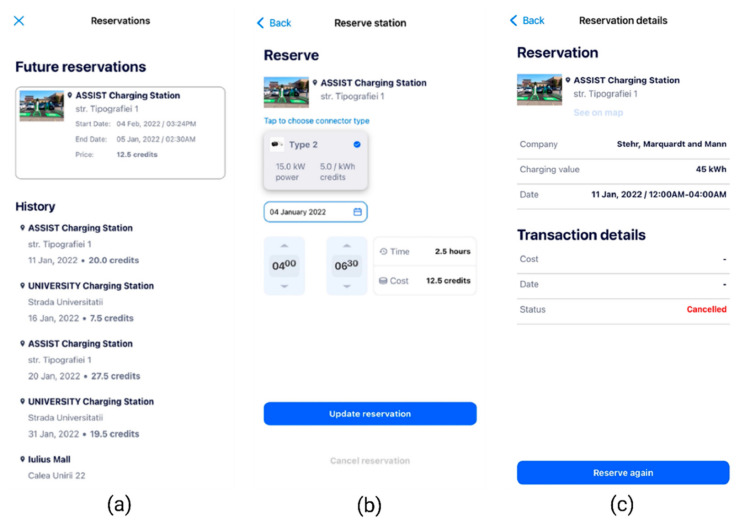
Smart EVC—Future reservations visualization. (**a**) Reservations history; (**b**) Update reservation screen; (**c**) Visualisation of a past reservation details, with possibility to reserve again.

The users are also able to view their future reservations and update a reservation if necessary (Figure 9a,b).

If the user updates their reservation with a new set of data, by clicking the “Update reservation” button (Figure 9b), the Reservation Algorithm will be called upon one more time and it will recalculate the response based on the newly entered data.

All the actions that are performed by the driver are used for the statistics, to ensure a better estimation and user experience when using the Reservation Algorithm.

The user is also allowed to cancel a reservation (Figure 9b). This action will release the occupied time slot from the reservation history and will also update the statistics used for other upcoming reservations. The reservation history will enable users to reserve the same charge point repeatedly (Figure 9c). This feature is convenient especially if the user travels on the same route frequently.

## 6. Results and Discussion

We performed several test cases for the reservation algorithm where the connector of the station is reserved for the entire day and there were no overlaps. Even if the station is disconnected and comes back online, only the user that has the reservation at that moment can start a transaction. Another example of a test case is when a driver finishes a transaction before the reservation time ends, in which case, the connector would be released, so other drivers can charge until the next reservation time arrives.

There are several external variables, though, that can influence the charging time and need to be further analysed, such as traffic jams, battery condition, energy consumption, and weather conditions.

• Traffic jams

Research made in 2021 by “Which?”, a United Kingdom organization that promotes informed consumer choice in the purchase of goods and services by testing products, has shown that in just over an hour and 15 min, 2% of battery from a 77 kWh battery was discharged. This is the equivalent of 13 km of range, considering it was on a summer day, but it is a well-known fact that cold weather will have a bigger impact on the car’s power usage [26].

• Battery condition

When the battery is new, it seems to last forever and charge quickly, but over time and use, its capacity will decline, and its charging time will increase. Even if the loss is not that big and is only around 2.3% per year, it is still significant when considering a charging reservation [27].

• Weather

A study on electric vehicles charging in cold temperatures has shown that drivers still face some charging issues. The reason is that cold temperatures have a significant impact on the whole charging process because of the electrochemical reactions, and the management system will limit the charging rate, to avoid battery damage. This is the reason for which there will be inevitable alterations in the charging time [28].

• Battery energy consumption

The energy consumption is defined in terms of kilowatt hour per one hundred kilometres, and it will obviously differ on a case-by-case basis and depending on many other factors such as driver’s behaviour or interior temperature regulation, but in the end, it will optimize or weaken the electricity consumption, which will certainly affect the charging time as well [29].

## 7. Conclusions

In this article, we proposed a solution for the challenge of electric vehicle reservations. We assumed that several users were waiting in a queue at a charging station with a single connector when following the OCPP reservation protocol that allowed for a lot of overlaps to occur. To implement a solution, we built an algorithm that creates reservation intervals based on coefficients provided from the reservation and transaction history of the specific charging station, ensuring consumer satisfaction. In addition, we changed the reservation perspective by adding the charging time to it. To solve the overlap problem, every reservation can be controlled by the user that created it so no one else can start a transaction in the reservation interval. Moreover, when the reserved time ends the charging stops. By taking this into a real scenario there will be no overlaps.

Our solution is a new addition to the EV charging reservation feature, as it ensures a separation of the reservation logic in the central system. This allows for a more efficient management of the authentication and reservation processes. In our system the reservation is made up of the selected charging time and the additional time is added before the actual charging transaction, as calculated by the algorithm. In comparison to other reservation algorithms, our solution focuses on the integrity of the central systems that serve as a mediator between the driver and the charging station. The drivers can check the charging stations’ available time slots, decide which times suit them best, and make the reservation, being assured that the connector will become unavailable to any other incoming EVs for the entire duration of the reservation. One limitation of the proposed solution is that users must currently enter the battery level manually and it is not automatically retrieved from the vehicle. Furthermore, our study relies on the assumption that they correctly estimate the vehicle’s battery level at the start of the planned journey.

## 8. Future Research Directions

To further expand the functionalities of the reservation module, we plan to add a new option where drivers can select a time span, and the application returns the closest charging points that are available at that moment.

To improve the algorithm, we intend to add a new method that generates an ideal reservation interval based on the current battery level and the level the user intends to reach at the end of the charging cycle. This involves taking into consideration all the influencing factors mentioned above and adding extra time to the reservation.
